# Editorial: Cell stress responses and metabolic reprogramming in skin diseases

**DOI:** 10.3389/fcell.2023.1171812

**Published:** 2023-03-01

**Authors:** Emanuela Bastonini, Daniela Kovacs, Fiorenza Lotti, Luis Sanchez-Del-Campo

**Affiliations:** ^1^ Laboratory of Cutaneous Physiopathology and Integrated Center of Metabolomics Research, San Gallicano Dermatological Institute, IRCCS, Rome, Italy; ^2^ Department of Experimental Oncology, IEO, European Institute of Oncology IRCCS, Milan, Italy; ^3^ Department of Biochemistry and Molecular Biology, School of Biology, IMIB-University of Murcia, Murcia, Spain

**Keywords:** skin diseases, melanoma, energetic metabolism, metabolic rewiring, cell metabolism, phenotype switching

Despite being in the challenging age of personalized and precision medicine, specific responses to external stressors may ultimately converge on common cell reprogramming mechanisms belonging to heterogeneous pathologies that range from metabolic disorders to cancer. Cell metabolism plays a crucial role in all living organisms and it is closely intertwined with multiple biochemical pathways, which should not simply viewed as a means to an end, since metabolic intermediates themselves can have huge functional importance. It is now recognized that there are many more metabolites, acting as signaling regulators than genes in the human genome. Perturbations of cell metabolism play an important role in both health and disease and perhaps their causative role is still underestimated. Certainly, some of those pathophysiological dysfunctions can result in cutaneous disorders.

The skin is the largest organ of the body and, as our first line of defense against the outside world, it can fall susceptible to infections, wounds, burns and tumors given its direct exposure to external threats. How cutaneous cells cope with the insults and finely coordinate stress responses to maintain fundamental physiological processes such as effective homeostasis, senescence, DNA repair, immunity and regeneration are key Research Topic. Moreover, skin is the only organ that gives us a daily visible “clinical picture” of our physiological state, also reflecting pathological conditions that may be occurring internally. Due to age, health and environment, skin is among the most dynamic, ever-changing organs of our body. Consequently, some stress-activated signalling pathways involving energy metabolism, mitochondrial functions and dynamics, inflammation, autophagy and cell death are impaired in many skin disorders.

Emphasis is now increasingly placed on understanding how the nature of skin is far more complex if viewed from a cell metabolism perspective and interest in the reciprocal regulation of stress responses and metabolism has extensively grown over the last decade. Metabolism has traditionally been seen as a downstream effector in the regulatory architecture of skin cells. Instead, the emerging concept is that metabolism commands cellular physiology, meaning that any cell change is linked to a metabolic adaptation.

In melanoma, the most aggressive type of skin cancer, a metabolic rewiring has been postulated to be a conserved cellular stress response. The distinct intra-tumor phenotypic heterogeneity of melanoma cell subpopulations ranging from differentiated, proliferating, invasive until intermediate states, may derive from cell-intrinsic stressed not only caused by the activation of oncogenes but also by non-genetic microenvironmental influences including hypoxia, nutrient limitation and response to therapy. A mutational signature may not be sufficient to explain either phenotypic heterogeneity or metastatic dissemination, since primary tumors and metastases often share the same mutational burden. On the contrary, different phenotypic states exhibit distinct metabolic profiles. New insights into metabolic remodelling occurring in cells during the phenotype switch revealed signalling pathways and/or specific metabolites that trigger cell fate transitions and target specific phenotypic states, independently of genotype. Furthermore, metabolic plasticity is crucial for the control of stem cell homeostasis by regulating the ability to differentiate into specific cell lineages.

Hence, as the greek origin of the word metabolism (“μεταβολη”) suggests, acting on dysregulated cell metabolism signalling networks may help to “change” disease outcomes.

Many microenvironmental triggers acting as stress injuries, such as glutamine limitation, hypoxia or inflammation, can induce, affect, or even exacerbate the onset and/or relapse of skin cancers through a translational reprogramming. In the Research Topic “*Cell stress responses and metabolic reprogramming in skin diseases*”, Guendisch et al. demonstrate that the transcription factor Yin Yang 1 (YY1), whose function is strictly related to the cell type, the context of its expression and the presence of co-factors, orchestrates metabolic pathways crucial for primary melanoma establishment while inhibiting its metastatic spreading. Loss of YY1 drives melanoma cell migration and invasion *in vitro* and *in vivo* inducing an invasiveness gene signature by activation of a metabolic stress program. Therefore, YY1 knockdown may mimic a melanoma cell-specific response to metabolic stress-induced by perturbance of YY1-related pathways in response to extrinsic triggers. Interestingly, YY1 evokes the reacquisition of neural crest stem cell (NCSC)-like characteristics in melanoma, the developmental structure which gives rise to melanocytes and melanoma.

In line with these results, the Research Topic includes a review article by Falletta et al., which highlights recent findings, advances and challenges supporting the emerging and fascinating concept that stress-activated metabolic plasticity, which allows prompt and reversible adaptation to microenvironmental conditions, may determine phenotypic identity in melanoma. In particular, they describe the nature of some events supporting metabolic plasticity. Beyond well established metabolic alterations mediated by oncogenic BRAF and metabolic rewiring governed by the transcription factor MITF, the authors focus on the role of Fatty Acid (FAs) metabolism in determining melanoma aggressiveness.

Recent advances in technologies including tandem mass spectrometry, RAMAN scattering microscopy and electrospray ionization revealed diagnostic lipid signatures in tumors. Among the most prominent metabolic reprogramming features in melanoma, there is the increased rate of lipid synthesis which has been associated with drug resistance in cellular models. Through the analyses of transcriptomic profiles and lipid configuration in melanoma resistant cells, Vergani et al. reveal dysregulation of lipid metabolism and composition, thus mapping distinct lipid fingerprints for sensitive and BRAFi-resistant cells. Thus, continuous efforts are being made to adjust lipid metabolism as anticancer drugs.

The skin, which is continuously exposed to physical injury, pathogens and UV exposure immediately activates stress-induced signalling to maintain its homeostasis. Epigenetic regulation of stress responses has been intensively studied in the skin. However, only more recently, small non-coding RNAs, derived from tRNA transcription, modification and/or fragmentation and roughly classified in tiRNAs (tRNA-derived stress-induced RNAs) and tRFs (tRNA-derived fragments) have emerged as new powerful players in the response to various stress signals, like UV irradiation. Fang and co-workers screen the expression profile of tiRNAs and tRFs in UVB-damaged skin and reveal that the genes specifically targeted and regulated by the small non-coding RNAs are significantly enriched in the Wnt signaling pathway, which is crucial in tumor metabolic reprogramming and inflammatory changes.

The range of Research Topic covered in the Research Topic entitled “*Cell stress responses and metabolic reprogramming in skin diseases*” highlights the new insights and rapid progresses made over recent years in our understanding of stress-activated signalling pathways in the skin. The Research Topic lends weight to the recent concept that metabolic plasticity may form a unique fingerprint for melanoma cells, depending on the cellular content and genetic, epigenetic, and microenvironmental alterations. The results generated to date and in the future will make a major contribution to address the outstanding questions and expand the therapeutic management of skin diseases. Learning to “read” our skin correctly and knowing it thoroughly, as well as how it acts under what circumstances may help to recognize genetic and non-genetic duality of the response to hostile environmental conditions.

